# Perceived rivalry in intergroup conflict

**DOI:** 10.3389/fpsyg.2026.1875356

**Published:** 2026-08-04

**Authors:** David A. Reinhard, Peter T. Gianiodis, Gilad Hirschberger, Bernhard Leidner

**Affiliations:** 1 Graduate School of Business, Nazarbayev University, Astana, Kazakhstan; 2 Palumbo-Donahue School of Business, Duquesne University, Pittsburgh, PA, United States; 3 Baruch Ivcher School of Psychology, Reichman University, Herzliya, Israel; 4 Psychology of Peace and Violence Program, University of Massachusetts Amherst, Amherst, MA, United States

**Keywords:** collective identity, enmity, intergroup conflict, international relations, rivalry

## Abstract

We draw on an emerging psychological view of rivalry as a relational perception and apply it to intergroup conflict. We examine whether perceived intergroup rivalry predicts support for conflict escalation above and beyond enemy-related perceptions and threat, and whether rivalry is more closely linked to conflict-related identity relevance, defined as the extent to which conflict with an adversary is seen as part of collective identity. Using a quota-based sample of Jewish Israelis approximating national representativeness, we assessed perceptions of Iran as a rival and as an enemy, support for conflict escalation, conflict-related identity relevance, and multidimensional threat. Perceived rivalry uniquely predicted support for conflict escalation. Perceived rivalry was also more closely associated with conflict-related identity relevance than enemy-related perceptions were, suggesting that rivals may be viewed as more deeply embedded in collective identity. These findings suggest that perceived intergroup rivalry captures a psychologically embedded form of adversarial perception that may help explain why some intergroup conflicts are especially difficult to defuse.

## Introduction

Intergroup conflict remains a persistent feature of social and political life. In international relations, conflict is concentrated among a relatively small set of adversarial relationships rather than distributed evenly across all possible opponents (Colaresi and Thompson, 2002). This concentration appears to have increased over time, suggesting that conflict has become progressively more clustered within entrenched adversarial relationships (Thompson and Dreyer, 2012). Many such relationships persist across generations and come to be viewed as difficult to resolve (Bar-Tal, 2000, 2007; Coleman et al., 2014). In such contexts, understanding how people view these adversaries is important for explaining support for further conflict escalation. Here, we examine whether perceived rivalry captures an aspect of adversarial relations that extends beyond enemy-related perceptions and threat, with implications for collective identity and support for escalation.

Boulding (1956, 1959) argued that people’s views of strategic choices toward other nations are shaped by the cognitive, affective, and evaluative images they hold of their own nation and of other nations. Building on this insight, image theory proposes that perceptions of other nations are organized into relatively coherent images shaped by the perceived character of the international relationship. In turn, these images help guide or justify preferences for particular foreign policy responses (Alexander et al., 1999; Alexander et al., 2005a; Cottam, 1977; Herrmann et al., 1999; Herrmann et al., 1997). The enemy image has traditionally garnered the most attention. It is associated with perceptions of threat (Li et al., 2016) and is especially important because it can fuel cycles of conflict escalation that become difficult to interrupt once underway (Bronfenbrenner, 1961; Jervis, 1976; Osgood, 1962). Portraying an outgroup as an enemy may also serve a functional purpose by weakening moral inhibitions against aggression and helping justify preemptive action against a target perceived as likely to attack first (Alexander et al., 1999).

The centrality of the enemy image also fits with broader research on intergroup threat, which shows that outgroups may be seen as threatening the ingroup’s safety and material wellbeing, as well as its values and way of life (LeVine and Campbell, 1972; Sherif and Sherif, 1953, 1979; Stephan and Stephan, 2000). Extending this multidimensional view of threat, Hirschberger et al. (2016) show that existential threat includes distinct forms that are linked to support for escalatory policies in intergroup conflict. More broadly, perceived threat has been shown to foster more negative outgroup attitudes (Riek et al., 2006) and greater support for hostile responses toward adversarial outgroups (Berinsky, 2007; Paez and Liu, 2011; Rozmann, 2025). Although this research tradition has been highly valuable, it leaves open whether some adversarial relationships are perceived in ways that are not fully reducible to threat and negative evaluation alone. This makes threat an important alternative explanation to account for when examining whether rivalry captures a distinct relational dimension of adversarial perception. In enduring conflicts, an adversary may be seen not only as threatening, but also as a recurring counterpart whose relationship with the ingroup becomes psychologically meaningful over time.

We argue that this relational dimension of enduring conflict is what rivalry is especially well suited to capture. For such relationships, rivalry may complement enemy-related perceptions by more directly reflecting the relational and identity-relevant features of adversarial relationships that extend beyond hostility or threat. Rivalry often arises through a history of notable opposition, particularly when opponents are geographically proximate, relatively evenly matched, and repeatedly engaged with one another (Kilduff et al., 2010). Unlike competition, which is defined by a structural arrangement in which parties’ goals are negatively linked (Deutsch, 1949), rivalry exists psychologically as a relational schema in which actors are bound by a script that connects past, present, and anticipated future encounters within a broader narrative (Converse and Reinhard, 2016). Because this schema can shape how actors understand themselves in relation to an adversary, rivalry may carry implications for identity and self-concept (Converse et al., 2024). Consistent with this view, prior work has linked rivalry to self-worth (Kilduff et al., 2016). These features suggest that rivalry may be especially relevant in enduring intergroup conflict, where repeated antagonism may become tied to collective memory and group narratives.

We refer to this as conflict-related identity relevance: the extent to which a group’s conflict with another group is seen as part of its own identity, history, and collective memory. The underlying premise is that recurring relationships with particular adversaries can become part of how people understand themselves. This premise draws on research on close relationships, which has emphasized how significant others can become incorporated into the self-concept (Baldwin, 1992; Chen et al., 2006). This idea also builds on social comparison research suggesting that enduring comparison groups can become important for defining the ingroup itself (Festinger, 1954), as well as recent work showing that some outgroups become so identity-relevant that changes to the relationship can alter how the ingroup understands itself (Berendt et al., 2024). More broadly, social psychological work suggests that self-conceptions are shaped by reference groups (Manis, 1955; Rosenberg, 1973) and can be organized at individual, relational, and collective levels (Brewer and Gardner, 1996).

Extending this logic to the intergroup domain, we suggest that adversarial intergroup relationships may also become identity-relevant at the collective level, particularly when they are repeated and historically meaningful. This extension is consistent with research suggesting that conflict-related narratives can become incorporated into group members’ identities (Hammack, 2008). It also aligns with work on identity and intractable conflict, which shows how such conflicts can involve identity dynamics and collective meaning (Northrup, 1989), the role of adversarial others in national self-understanding (Kelman, 1999), conflict-supporting narratives (Oren et al., 2015), and shared representations of historical suffering (Volkan, 2001). Rivalry is likely to correspond to conflict-related identity relevance because it implies repeated engagement and shared conflict history, rather than hostility alone. When a conflict is tied to collective identity, history, and memory, the adversary may be viewed less as an interchangeable source of threat and more as a recurring counterpart in a relationship that matters to the ingroup. This perception may, in turn, help explain support for escalation by making continued conflict appear connected to the preservation of a meaningful adversarial relationship. Enemy perceptions, by contrast, place greater emphasis on threat and negativity toward an outgroup than on the repeated and embedded character of the adversarial relationship itself.

Rivalry may therefore better capture adversarial relationships that have become psychologically embedded in a group’s collective identity. Although rivalry and enemy perceptions emerge from distinct literatures, the key question here is whether that distinction matters for explaining support for conflict escalation and its connection to conflict-related identity relevance. This logic is consistent with image theory, which treats images of other groups as organized representations of intergroup relationships that are shaped by social identification with the ingroup and that, in turn, help justify policy preferences toward those groups (Alexander et al., 2005b; Herrmann et al., 1997). For conflict escalation support, rivalry may complement enemy-related perceptions because escalation can arise from multiple ways of construing an adversarial relationship. For conflict-related identity relevance, however, rivalry may be more theoretically aligned because it entails a recurring relationship with a particular adversary. Accordingly, we argue that rivalry reflects a relational schema not reducible to threat or enemy-related perceptions alone. To evaluate these ideas, we examine whether perceived intergroup rivalry is uniquely associated with support for conflict escalation in the context of international conflict, whether it is more closely linked to conflict-related identity relevance than enemy-related perceptions are, and whether it statistically accounts for the association between this identity-relevant dimension and support for escalation.

### Overview of the current study

The Israel-Iran relationship offers a useful context for testing the present arguments because it is widely perceived as a serious and enduring adversarial relationship marked by repeated political, military, and proxy-based opposition over time (for an overview of the conflict, see Kaye et al., 2011). This makes it a suitable case for examining whether support for conflict escalation is associated not only with enemy-related perceptions and threat, but also with perceived rivalry and its link to conflict-related identity relevance.

## Method

### Participants

We recruited Jewish Israeli adults through the Midgam Project,^1^ an Israeli survey research firm, using age and gender quotas to approximate national representativeness. The final sample included 307 participants who completed the survey. Power and sensitivity analyses are reported in the Supplemental Material (SM).

### Measures

All continuous measures used 9-point Likert slider scales (e.g., 1 = not at all to 9 = very much) that recorded responses to the hundredth decimal. We computed multi-item composites by averaging their items after reverse-scoring relevant items. See the SM for the full survey items, including both English and Hebrew versions, and additional scale-construction details.

#### Conflict escalation

Conflict escalation support was measured using a five-item scale adapted from prior research (Golec and Federico, 2004). An example item was “Demonstrate strength in order to intimidate Iran.” Participants responded using a 9-point Likert scale, ranging from 1 (very opposed) to 9 (very supportive). The resulting scale demonstrated good internal consistency (*α* = 0.79).

#### Perceived rivalry

Perceived rivalry was measured using a modified scale adapted from prior research (Converse and Reinhard, 2016; Kilduff, 2014). This measure consisted of four items. An example item was “As an Israeli, do you feel a rivalry with Iran?” Participants responded using a 9-point Likert scale, ranging from 1 (not at all) to 9 (very much). The resulting scale demonstrated adequate internal consistency (*α* = 0.69).

#### Enemy perceptions

To assess enemy perceptions, we included two indicators: an enemy image scale adapted from prior research and an exploratory enemy-label item.

*Enemy image*. To measure the enemy image, we used a modified version of an established scale adapted from prior research (Alexander et al., 2005a; Alexander et al., 2005b; Li et al., 2016). The measure initially included four items. An example item was “Iran’s objectives are self-centered and harmful to others.” The third item was reverse-scored so that higher values consistently reflected stronger enemy image perceptions. The fourth item, “Iran has a strict, well-organized authority structure for decision making,” was dropped due to low inter-item correlations with the remaining items. Participants responded using a 9-point scale ranging from 1 (strongly disagree) to 9 (strongly agree). The resulting scale demonstrated modest internal consistency (*α* = 0.63), comparable to prior uses of this enemy image measure (e.g., Alexander et al., 2005b).

*Enemy label*. In addition, participants completed a categorical item asking which label best characterized Iran in relation to Israel (ally, enemy, opponent, rival, or other—free response). We used responses to this item to construct an exploratory dummy-coded enemy-label variable (1 = enemy, 0 = otherwise).

#### Conflict-related identity relevance

Conflict-related identity relevance was measured using a four-item scale. An example item was “The conflict against Iran is a core part of Israel’s history and collective memory.” Participants responded using a 9-point scale ranging from 1 (strongly disagree) to 9 (strongly agree). The resulting scale demonstrated good internal consistency (*α* = 0.83).

#### Multidimensional existential threat

Threat was measured using a modified version of the Multidimensional Existential Threat scale (Hirschberger et al., 2016), which includes three dimensions: physical collective annihilation (PA), symbolic collective annihilation (SA), and past victimization (PV). Participants responded using a 9-point scale ranging from 1 (strongly disagree) to 9 (strongly agree).

*Physical collective annihilation (PA).* This dimension was measured using nine items. An example item was “The physical existence of the Israeli people is in danger if Israel allows Iran to move forward with its nuclear program.” The resulting scale demonstrated excellent internal consistency (*α* = 0.92).

*Symbolic collective annihilation (SA).* This dimension was measured using six items. An example item was “Israeli culture will always maintain its uniqueness from Persian culture.” Here, Persian culture was used to denote a broader cultural referent rather than the contemporary Iranian state. The resulting scale demonstrated good internal consistency (*α* = 0.81).

*Past victimization (PV)*. This dimension was measured using four items. An example item was “I tend to reflect on moments in Israeli history where Israel was under threat from Iran.” The resulting scale demonstrated acceptable internal consistency (*α* = 0.75).

#### Control variables

To consider alternative explanations, we also modeled a series of other variables, including political ideology (measured from 1 = left-wing to 9 = right-wing), social identification with Israel measured by reported levels of glorification (e.g., “Relative to other nations, we are a very moral nation,” ranging from 1 (strong disagree) to 9 (strongly agree), Roccas et al., 2006), age, gender, and education. We expected these characteristics would be associated with conflict escalation support and accounted for them in the subsequent analyses.

### Procedure

#### Data collection

This research was approved by the institutional review board (IRB) of the Interdisciplinary Center Herzliya. Before beginning the study, participants provided electronic informed consent. They completed the survey in Hebrew on October 6, 2019, received a small monetary reward for participation, and were subsequently debriefed.

#### Data analysis

We first examined zero-order associations using Pearson bivariate correlations. We then conducted a series of multiple linear regressions using ordinary least squares (OLS) to examine rivalry’s unique association with conflict escalation support. We then used Hayes’s SPSS PROCESS procedure (Hayes, 2017). We estimated a parallel mediation model (Model 4) in which rivalry and enemy image were specified as statistical mediators of the association between conflict-related identity relevance and conflict escalation support, with threat dimensions included as covariates to account for threat-related alternatives. Indirect effects were estimated using 10,000 bootstrap samples and 95% confidence intervals. Given the cross-sectional nature of the data, we also estimated a supplementary model reversing the placement of rivalry and conflict-related identity relevance.

## Results

Bivariate correlations provided an initial descriptive view of associations among the study variables. Perceived rivalry was positively associated with both enemy-related perceptions (correlation coefficients ranged from 0.33 to 0.42), suggesting that these perceptions overlap but may not be interchangeable. Consistent with that possibility, rivalry was positively associated with conflict-related identity relevance, whereas neither enemy image nor the exploratory enemy label measure was significantly related to this construct. This pattern is consistent with the view that conflict-related identity relevance is more closely linked to rivalry than to enemy-related perceptions more broadly. Direct comparisons using Williams’s tests supported this interpretation: conflict-related identity relevance was more strongly associated with rivalry than with enemy image, Williams’s *t*(302) = 6.09, *p* < 0.001, and with the exploratory enemy-label indicator, Williams’s *t*(302) = 4.23, *p* < 0.001.

Support for conflict escalation was positively associated with rivalry, enemy image, the exploratory enemy label measure, and conflict-related identity relevance. Table 1 reports the bivariate correlations, and Supplementary Table S1 reports descriptive statistics for all study variables. Several significant correlations were small-to-moderate in magnitude, and no multiple-testing correction was applied because focal tests were reserved for later models. We next turned to multiple regression analyses to assess whether rivalry predicted support for conflict escalation above and beyond related adversarial perceptions.

**Table 1:** Correlations for study variables.

Variable	1	2	3	4	5	6	7	8	9
1. Rivalry	–								
2. Enemy image	0.42***	–							
3. Enemy label^a^	0.33***	0.40***	–						
4. Identity relevance^b^	0.34***	−0.01	0.07	–					
5. MET: PA	0.42***	0.41***	0.34***	0.23***	–				
6. MET: SA	0.25***	0.39***	0.19***	0.10	0.22***	–			
7. MET: PV	0.33***	0.23***	0.25***	0.40***	0.35***	0.32***	–		
8. Glorification	0.29***	0.44***	0.27***	0.22***	0.34***	0.52***	0.39***	–	
9. Political ideology	0.28***	0.28***	0.17**	0.28***	0.34***	0.38***	0.30***	0.51***	–
10. Escalation	0.33***	0.26***	0.29***	0.16**	0.22***	0.30***	0.30***	0.35***	0.44***

MET = multidimensional existential threat. *p < 0.05, **p < 0.01, ***p < 0.001.

a Dummy-coded (1 = Yes, 0 = No).

b Conflict-related identity relevance.

To assess whether rivalry was uniquely associated with support for conflict escalation, we estimated hierarchical OLS regression models in which control variables and related adversarial perceptions were entered first, followed by rivalry. Model 1 included enemy image, enemy label, the MET dimensions, glorification, political orientation, and demographic fixed effects; Model 2 added rivalry. In Model 2, rivalry was positively associated with support for conflict escalation (*β* = 0.17, *p* = 0.002), indicating that rivalry explained unique variance beyond the other included predictors. Adding rivalry significantly improved model fit, *F* = 9.48, *p* = 0.002, increasing *R^2^* from 0.305 to 0.327 (Δ*R^2^* = 0.022, Cohen’s *f*^2^ = 0.033). The enemy label remained a significant predictor in the full model, whereas enemy image did not. Full results for Models 1 and 2 are reported in Table 2.

**Table 2:** OLS multiple regression for conflict escalation measure.

Independent variables	Model 1	Model 2
Rivalry	–	0.17** (0.06)
Enemy image	0.02 (0.06)	−0.02 (0.06)
Enemy label	0.17** (0.06)	0.15** (0.06)
MET: physical annihilation	−0.01 (0.06)	−0.05 (0.06)
MET: symbolic annihilation	0.09 (0.06)	0.08 (0.06)
MET: past victimization	0.11* (0.06)	0.08 (0.06)
Glorification	0.03 (0.07)	0.04 (0.07)
Political orientation	0.32*** (0.06)	0.30*** (0.06)
Age fixed effect included	Yes	Yes
Gender fixed effect included	Yes	Yes
Education fixed effect included	Yes	Yes
*R* ^2^	0.3054	0.3274
Δ*R*^2^	–	0.0220
Adjusted *R*^2^	0.2816	0.3019
DF	291	290

*p < 0.05, **p < 0.01, ***p < 0.001. Entries are standardized regression coefficients with standard errors in parentheses. Coefficients for age, gender, and education are omitted for brevity.

**Figure 1:**
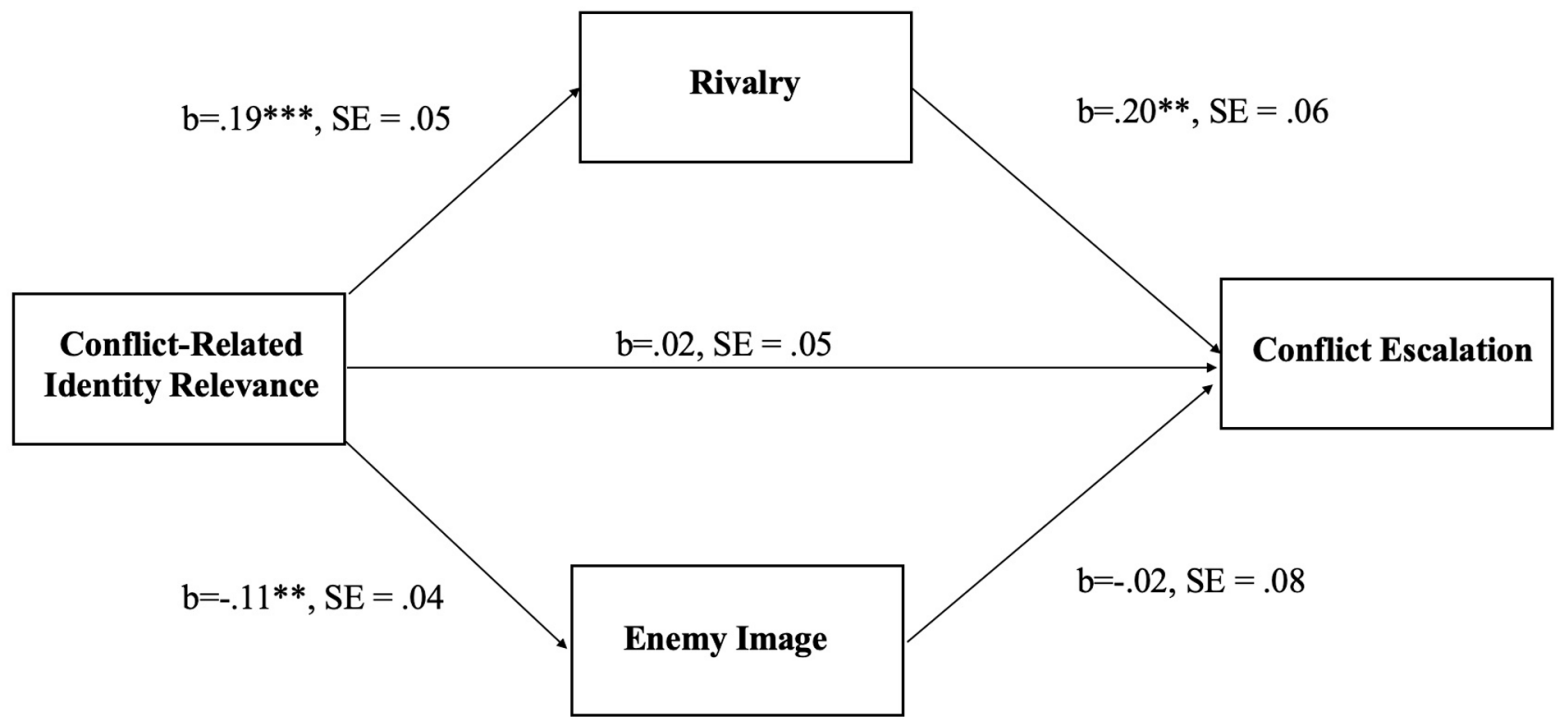
Parallel mediation of conflict-related identity relevance on conflict escalation by rivalry and enemy image. Bootstrap confidence interval for rivalry: [0.01, 0.07]. Bootstrap confidence interval for enemy image: [−0.02, 0.02]. Gender, age, education, political orientation, glorification, enemy label, and MET: PA, SA, PV as covariates. *p < 0.05, **p < 0.01, ***p < 0.001.

Because rivalry remained uniquely associated with support for conflict escalation in the multivariate models, we next tested a parallel mediation model in which rivalry and enemy image were specified as statistical mediators of the association between conflict-related identity relevance and support for conflict escalation. As shown in Figure 1, conflict-related identity relevance was positively associated with rivalry, and rivalry was positively associated with support for conflict escalation, after controlling for gender, age, political orientation, education, glorification, enemy label, and the MET dimensions. The indirect effect via rivalry was significant (*b* = 0.04, *BootSE* = 0.017, 95% CI [0.01, 0.07]). Although statistically significant, this modest indirect association is theoretically informative because it emerged after accounting for enemy image, the exploratory enemy-label indicator, the MET dimensions, and additional covariates. Conflict-related identity relevance was also negatively associated with enemy image. Notably, this negative coefficient emerged despite the near-zero bivariate correlation between conflict-related identity relevance and enemy image and should therefore be interpreted as a conditional association after accounting for rivalry and the included covariates. Enemy image was not significantly associated with support for conflict escalation. Consistent with this, the indirect effect via enemy image was not significant (*b* < 0.01, *SE* = 0.01, 95% CI [−0.02, 0.02]). A bootstrapped contrast indicated that the indirect effect through rivalry was larger than the indirect effect through enemy image, contrast = 0.036, *BootSE* = 0.018, 95% CI [0.002, 0.074].

Together, these findings are consistent with the proposed indirect association between conflict-related identity relevance and support for conflict escalation via rivalry, but not via enemy image. In a supplementary model reversing the placement of rivalry and conflict-related identity relevance, the indirect effect was not significant (see Supplementary Figure S1).

## Discussion

The study examined whether perceived rivalry provides a distinct pathway through which conflict-related identity relevance is associated with support for conflict escalation, beyond enemy-related perceptions and threat. Our findings suggest that perceived rivalry captures an aspect of adversarial perception in intergroup conflict that is not fully reducible to enemy-related perceptions or threat. Rivalry instead appears to reflect a more relational and identity-relevant way of construing an adversary. Consistent with this interpretation, rivalry predicted support for conflict escalation above and beyond enemy-related perceptions and threat. Further, rivalry was more closely associated with conflict-related identity relevance than enemy-related perceptions were, and statistically accounted for the association between this construct and support for escalation.

By contrast, enemy image did not significantly mediate this association, suggesting that conflict-related identity relevance was more closely aligned with rivalry than with enemy image perceptions. The negative association between conflict-related identity relevance and enemy image in the mediation model should be interpreted cautiously because it emerged only after accounting for rivalry and covariates, whereas the bivariate association was near zero. Likewise, enemy image was associated with escalation support bivariately but not in the mediation model, suggesting that its association with escalation may overlap with rivalry, threat, and other covariates. Once identity-relevant features shared with rivalry are accounted for, enemy image may capture a more purely negative perception of the adversary rather than a relationship experienced as part of collective identity.

### Implications

These findings help clarify the theoretical role of rivalry in intergroup conflict. They extend research on image theory in general, and enemy images in particular, by suggesting that hostility and threat may not fully capture how some enduring adversarial relationships are psychologically represented. Specifically, rivalry may capture an additional relational dimension of adversarial perception. This is important because in some conflicts, the adversary may be viewed not only as dangerous or immoral, but also as a recurring and identity-relevant counterpart. This conceptualization of conflict-related identity relevance also enriches traditional accounts of social identity, which have predominantly focused on the ingroup (Tajfel, 1974), by suggesting that outgroups may matter not only as objects of comparison, but also as relationships woven into collective identity. In enduring conflicts, the rival may become part of how group members make sense of their group’s history, continuity, and identity. Future research could also extend this perspective by connecting it to adjacent work on identity, including self-expansion (Aron et al., 2013), disidentification (Elsbach and Bhattacharya, 2001), and negational identities (Zhong et al., 2008), to clarify how adversarial relationships may shape collective identity in different ways.

These findings also have practical implications for conflict resolution. Much prior research explains support for conflict in terms of perceived threats, including threats to distinctiveness, group esteem, or collective existence (e.g., Brewer, 1991; Ellemers et al., 2002; Hirschberger et al., 2016). The current research adds a complementary perspective by suggesting that support for escalation may also persist when an adversarial relationship is experienced as a rivalry and becomes tied to collective identity. When rivalry becomes identity-relevant, resolving conflict may be experienced not only as the removal of danger, but also as the disruption of a meaningful adversarial relationship. Changes in a rival relationship may therefore raise questions about collective identity, continuity, and self-definition. Although the present study does not directly test such processes, this possibility is consistent with Bar-On’s (2008) argument that ending conflict may require groups to reconstruct aspects of how they understand themselves. These findings therefore raise the possibility that some adversarial relationships are difficult to relinquish not only because they are threatening, but also because they become tied to collective identity. Future research could examine whether declining rivalry is associated with perceived identity disruption, uncertainty, or resistance to reconciliation. It could also examine potential mechanisms through which groups may sustain a fading rivalry or redirect that identity-relevant antagonism toward a replacement rival.

Finally, these findings have broader theoretical and practical implications for how rivalry is studied and understood. Because rivalry is a perceived relationship, it is unlikely to be static or uniform. The meaning of an international relationship may shift over time in response to political events, historical developments, and changing collective narratives. Likewise, rivalry may be experienced differently across levels of society, including among individuals, organizations, and regions. Understanding rivalry therefore requires attention not only to objective conflict history, but also to how people construe the relationship and which aspects of that history are psychologically salient. This matters because public opinion can influence how people think governments can and should respond to international conflict (Converse and Traugott, 1986; see also Kteily et al., 2016; O’Brien et al., 2017). It is therefore important to examine not only material interests and strategic conditions, but also how people psychologically construe adversarial relationships.

### Limitations

Several limitations should be noted. First, the data are cross-sectional, which limits causal inference. Although the mediation analyses are consistent with the proposed model, they cannot establish temporal or causal order. Future research should examine these relationships using longitudinal or experimental designs. Second, the rivalry and enemy image measures showed only modest internal consistency, particularly for enemy image, suggesting that future work would benefit from further measurement refinement in international conflict contexts. Third, the study focused on one enduring conflict involving Jewish Israelis’ perceptions of Iran. Additional research is needed to determine whether similar patterns emerge in other intergroup and international rivalries, including rivalries that differ in whether conflict is experienced directly or through proxy groups, and in whether adversaries are geographically contiguous or more spatially distant. Despite these limitations, the present findings suggest that understanding support for conflict escalation may require attention not only to enemy perceptions, but also to rivalry as a psychologically embedded, identity-relevant form of adversarial perception.

## Conclusion

The present research applies a social-cognitive view of rivalry (Converse and Reinhard, 2016; Kilduff and Galinsky, 2017; Converse et al., 2024), grounded in relational schema theory (Baldwin, 1992), to the study of international conflict. Studying rivalry in this context may help conflict research better capture forms of adversarial perception that are not fully reducible to enemy perceptions or threat alone, and may offer a useful lens for understanding why some conflicts are especially resistant to de-escalation. In the present context, this perspective helps illuminate why perceived rivalry with Iran may matter for Israeli support for conflict escalation above and beyond enemy-related perceptions.

## Data Availability

The datasets presented in this study can be found in online repositories. The names of the repository/repositories and accession number(s) can be found in the article/Supplementary material.

## Footnotes

1
www.midgam.com
