# Supplementary material for: Perceived rivalry in intergroup conflict
Source: Front Psychol. 2026 Aug 4;17:1875356. doi: 10.3389/fpsyg.2026.1875356 (PMC13481545; doi:10.3389/fpsyg.2026.1875356)
Supplement: Supplementary file 1 [file Supplementary_file_1.DOCX]

Supplementary Material

# Sample-size Justification and Sensitivity Analyses

We targeted approximately 300 completed surveys, given evidence that correlation estimates in social science survey research often stabilize around 250 participants (Schönbrodt & Perugini, 2013). A sensitivity analysis indicated that this sample size provided 95% power to detect an effect size of r = .125 (Faul et al., 2007). To further align the sample-size justification with the primary hierarchical regression analysis, we also conducted a sensitivity analysis for the incremental contribution of rivalry. Using α = .05, power = .80, one tested predictor, and the residual degrees of freedom from the full model, *df* = 290, the analysis indicated that the model was sensitive to detect an incremental effect of *f²* = .027, equivalent to Δ*R²* = .018 given the full-model *R²*. The observed incremental contribution of rivalry was *f²* = .033, corresponding to Δ*R²* = .022. Thus, the sample was sensitive to an incremental effect of the observed magnitude for rivalry beyond enemy image, threat-related variables, and covariates.

**Procedure**

The order of the main questionnaires was randomized with the participants completing the demographic items at the end of the survey.

**Scale Construction and Missing Data**

We computed multi-item scale scores by averaging the items included in each scale. Items were reverse-scored where required by the scale structure or supported by the empirical item pattern. We report item exclusions in the relevant measure descriptions. We assigned participants a composite score only when they had valid responses for all items included in that scale; otherwise, we coded the composite as missing. Thus, the minimum number of valid items required equaled the number of items included in each scale. Item-level missingness was minimal across the focal measures. We used listwise deletion for the variables included in each analysis.

**OLS Diagnostics**

We examined OLS diagnostic indicators for the full hierarchical regression model reported as Model 2 in Table 2 of the main text. Variance inflation factors were low, ranging from 1.02 to 1.85, indicating no evidence of problematic multicollinearity. A Breusch-Pagan test did not indicate evidence of heteroscedasticity, *BP* = 15.30, *df* = 11, *p* = .169. Cook’s distance did not indicate highly influential cases, with a maximum value of .088. The focal rivalry effect remained significant when observations exceeding the conservative 4/n Cook’s distance threshold were excluded, *β* = .27, *SE* = .05, *p* < .001, and when using HC3 robust standard errors, *β* = .18, *SE* = .07, *p* = .009.

**Supplemental Mediation Analyses**

To aid interpretation of substantive magnitude, we also examined standardized mediation estimates. In standardized terms, conflict-related identity relevance was positively associated with rivalry, *β* = .22, and rivalry was positively associated with support for conflict escalation, *β* = .18. The completely standardized indirect effect through rivalry was small but statistically significant, *β* = .04, BootSE = .018, 95% CI [.01, .08]. The corresponding completely standardized indirect effect through enemy image was not significant, *β* < .01, BootSE = .01, 95% CI [−.02, .02].

## Supplementary Figures

***Figure S1***

*Parallel Mediation of Rivalry on Conflict Escalation by Conflict-Related Identity Relevance*

**Supplementary Figure S1.** Bootstrap Confidence Interval for Conflict-Related Identity Relevance: [-.05, .05]. Gender, Age, Education, Political Orientation, Glorification, Enemy image, & Enemy Label as covariates.

## Supplementary Tables

***Table S1***

*Descriptive Statistics for Study Variables*

| **Variables** | **Mean** | **Median** | **SD** | **Skewness** | **Kurtosis** | **Minimum** | **Maximum** | ***N*** |
| --- | --- | --- | --- | --- | --- | --- | --- | --- |
| 1. Rivalry | 6.18 | 6.35 | 1.58 | .34 | 2.48 | 1 | 9 | 307 |
| 3. Enemy image | 7.11 | 7.17 | 1.42 | -.52 | 2.86 | 2.18 | 9 | 307 |
| 4. Enemy label^a^ | .75 | N/A | .44 | -1.13 | 2.28 | 0 | 1 | 307 |
| 5. Identity relevance^b^ | 4.13 | 4.06 | 1.85 | .34 | 2.48 | 1 | 9 | 305 |
| 6. MET: PA | 6.36 | 6.52 | 1.95 | -.61 | 2.76 | 1 | 9 | 305 |
| 7. MET: SA | 6.07 | 6.15 | 1.00 | -.37 | 2.73 | 3.08 | 7.67 | 306 |
| 8. MET: PV | 5.41 | 5.37 | 1.66 | -.23 | 3.02 | 1 | 9 | 306 |
| 9. Glorification | 6.92 | 7.07 | 1.71 | -.67 | 3.02 | 1 | 9 | 305 |
| 10. Political ideology | 6.41 | 6.63 | 2.09 | -.48 | 2.38 | 1 | 9 | 305 |
| 11. Escalation | 5.34 | 5.19 | 1.71 | .21 | 2.75 | 1.17 | 9 | 306 |

*Note*. MET = Multidimensional Existential Threat.

^a^Dummy-coded (1 = Yes, 0 = No)

^b^Conflict-related identity relevance

# Questionnaires

Below, we report the questionnaire items for the focal measures included in the main-text analyses. The full survey instrument, including measures not analyzed in the present manuscript, is available at [[Link](https://osf.io/jxryd/overview?view_only=3e7d212c23e9496fb720b3f4e29bd82e)].

***Perceived Rivalry***

**1 כלל לא =**

**9**= **במידה רבה**

Read each item and indicate your response on the scale by moving the slider to the position that most accurately represents the way you feel.

**אנא קרא כל היגד וציין את תגובתך על ידי הזזת הסמן למיקום אשר מייצג בצורה המדויקת ביותר את תחושותיך.**

1. As an Israeli, do you feel a rivalry with Iran?

כישראלי, האם אתה מרגיש יריבות עם איראן?

2. As an Israeli, interactions between Israel and Iran are more important because of its rivalry with Israel.

כישראלי אני מרגיש שאינטראקציות בין ישראל לאיראן חשובות יותר בגלל יריבותה של איראן עם ישראל.

3. As an Israeli, I feel like the rivalry puts more on the line than just the objective stakes of the conflict with Iran.

כישראלי, אני חש כי ביריבות עם איראן יש יותר על כף המאזניים מאשר הנושאים האובייקטיבים של הסכסוך עמה.

4. As an Israeli, I view Iran as a rival of Israel.

כישראלי, אני רואה באיראן יריבה של ישראל.

***Enemy image***

**1 כלל לא מסכים=**

**9= מסכים במידה רבה**

Please indicate how much you agree or disagree with each of the statements below.

אנא ציין את מידת הסכמתך או אי הסכמתך עם כל אחד מההיגדים המופיעים מטה.

1. Iran's objectives are self-centered and harmful to others.

מטרותיה של איראן אנוכיות ומזיקות למדינות אחרות.

2. Iran would take advantage of any efforts on the part of others to cooperate, and it would even try to exploit others.

איראן תנסה להשתמש לרעה בכל מאמץ של אחרים לשיתוף פעולה ואפילו תנסה לנצל אותם.

3. Iran is extremely competitive and wants to dominate but will play by the rules.

איראן הינה מדינה תחרותית מאוד השואפת לשלוט, אך תפעל על פי חוקי המשחק.

4. Iran has a strict, well-organized authority structure for decision making.

באיראן יש תהליך קבלת החלטות מאורגן-היטב, סמכותני ומוקפד.

***Enemy Label (Enemy vs. Rival vs. Opponent vs. Ally)***

Which best characterizes Iran in relation to Israel?

מה מאפיין בצורה הטובה ביותר את איראן ביחסיה עם ישראל?

- Ally
- **בעלת ברית**
- Enemy
- אויבת
- Opponent
- מתנגדת
- Rival
- יריבה
- Other (fill in the blank)
- אחר_______

***Conflict-Related Identity Relevance***

1. The conflict against Iran is a core part of Israel’s history and collective memory.

**הסכסוך עם איראן הוא חלק מרכזי מההיסטוריה הישראלית ומהזיכרון של הציבור הישראלי.**

2. The shared history of conflict against Iran makes up an important part of the Israeli identity.

**ההיסטוריה של הסכסוך בין ישראל לאיראן מהווה חלק חשוב מהזהות הישראלית.**

3. The conflict with Iran plays a significant role in what it means to be Israeli.

**הסכסוך עם איראן ממלא תפקיד חשוב בהגדרה של מה זה אומר להיות ישראלי.**

4. The history between Iran and Israel should never be forgotten if we want to maintain Israeli identity.

**אם אנו רוצים לשמור על הזהות הישראלית, אסור שנשכח את ההיסטוריה בין ישראל ואיראן.**

5. Deemphasizing the role Iran played in Israeli history would significantly change what it means to be Israeli.

**המעטה בחשיבות של איראן בהיסטוריה הישראלית, תשנה משמעותית את ההבנה של מה זה להיות ישראלי.**

***Multidimensional Existential Threat***

On the next three pages, we are interested in your feelings regarding threats that Israelis face. There are many different types of threats that Israelis may face, including violence, threats to culture, and threats to the Israeli way of life. Please keep these in mind when responding to the statements on the next pages.

As the statements are about your feelings, again there are no right or wrong answers.

בשלושת העמודים הבאים, אנו מעוניינים להבין כיצד אתה מרגיש כלפי איומים שונים המאיימים על ישראלים. ישנו מגוון של איומים שונים שישראלים עשויים לחוות, הכוללים אלימות, איומים על התרבות הישראלית, ואיומים על אורח החיים הישראלי.

אנא הזכר באיומים אלו כאשר אתה עונה על ההיגדים בעמודים הבאים.

אנא זכור כי מכוון שהיגדים אלו עוסקים בתחושותיך, אין תשובות נכונות או שגויות.

**1 כלל לא מסכים=**

**9= מסכים במידה רבה**

Symbolic Annihilation:

1. Despite the tensions with Iran, the Israeli people have, and will continue to, live

prosperously.

למרות המתחים עם איראן, ישראלים ימשיכו לחיות בשגשוג ופריחה.

2. I believe that Israeli identity will always exist independently from Persian culture.

אני מאמין כי הזהות הישראלית תמיד תהיה קיימת כזהות נפרדת מזהויות אחרות.

3. Israeli culture will always prevail over Persian culture.

התרבות הישראלית לעולם תגבר על תרבויות אחרות סביבה.

4. Israeli culture will always maintain its uniqueness from Persian culture.

התרבות הישראלית תמיד תשמור על ייחודיותה מתרבויות אחרות סביבה.

5. Israeli culture will always be distinct from Persian culture.

התרבות הישראלית תמיד תבדל מתרבויות אחרות.

6. Eventually, Israelis will assimilate into other cultures.

בסופו של דבר הישראלים יתבוללו בתוך תרבויות אחרות.

Physical Annihilation:

1. The physical existence of the Israeli people is in danger if Israel allows Iran to move

forward with its nuclear program.

אם ישראל תאפשר לאיראן להתקדם בתכנית הגרעין שלה, הקיום הפיזי של הישראלים יהיה בסכנה.

2. If Israel allows Iran to further advance its nuclear program, Israel may soon find itself

on the verge of annihilation.

ישראל עלולה למצוא עצמה על סף השמדה בקרוב, אם תאפשר לאיראן להתקדם בתכנית הגרעין שלה.

3. The existence of the Israeli people is in jeopardy if Israel allows Iran to further advance its nuclear program.

אם ישראל תאפשר לאיראן להתקדם בתכנית הגרעין שלה, קיומו של העם הישראלי בסכנה.

4. The future of Israel looks bleak if Israel allows Iran to further advance its nuclear program.

העתיד הישראלי נראה עגום, אם ישראל תאפשר לאיראן להתקדם בתכנית הגרעין שלה.

5. Israel faces a threat to its existence if Israel allows Iran to further advance its nuclear program.

ישראל תעמוד בפני איום לקיומה, אם תאפשר לאיראן להתקדם בתכנית הגרעין שלה.

6. Israel will not survive for very long if Israel allows Iran to further advance its nuclear program.

ישראל לא תשרוד עוד זמן רב, אם תאפשר לאיראן להתקדם בתכנית הגרעין שלה.

7. There is no real threat to the existence of the Israeli people.

אין איום ממשי לקיומו של עם ישראל.

Past Victimization:

1. I often think of the suffering caused to Israelis in the past (e.g., AMIA bombing in Buenos Aires).

לעיתים קרובות אני חושב על הסבל שנגרם לישראלים בעבר

The negative treatment of Israelis in the past bothers me (e.g., the AMIA bombing in Buenos Aires).

מטריד אותי היחס השלילי שישראלים חוו בעבר

2. I tend to reflect on moments in Israeli history where Israel was under threat from Iran.

אני נוטה לחשוב על רגעים בהיסטוריה הישראלית, בהם ישראל הייתה תחת האיום של איראן.

4. I usually do not think about moments in Israeli history that involve conflict with Iran.

אני בדרך כלל לא חושב על רגעים בהיסטוריה, בהם ישראל הייתה מעורבת בסכסוך עם איראן.

***Political Conflict Resolution Questionnaire***

**1= מאוד מתנגד**

**9= מאוד תומך**

Now we would like you to think about different steps Israel could use in negotiations to end the conflict with Iran. Take a while to think over what sort of actions you might want Israel to undertake in these negotiations, and what sort of actions would be very unlikely for you to support them taking.

כעת אנו מעוניינים שתחשוב על המהלכים השונים שישראל יכולה לעשות במהלך משא ומתן לסיום הסכסוך עם איראן. אנא קח זמן לחשוב על איזה סוג פעולות ייתכן שתרצה שישראל תנקוט במהלך משא ומתן זה, ואילו פעולות סביר שלא תתמוך בהן.

On the next two pages you will find examples of various actions countries can assume in conflicts. Please indicate how likely it is that you would support for Israel to take a given action in negotiations with Iranian officials.

בשני העמודים הבאים תמצא דוגמאות למגוון פעולות שמדינות יכולות לנקוט בסכסוכים. אנא ציין עד כמה סביר שתתמוך בפעולות אלו במידה וישראל הייתה נוקטת בהם במהלך משא ומתן עם איראן.

Please choose your answers based on your thoughts regarding the conflict between Israel and Iran. Remember, there are no right or wrong answers.

אנא בחר את תשובותיך בהתבסס על מחשבותיך אודות הסכסוך בין ישראל לאיראן. זכור כי אין תשובות נכונות או שגויות.

Escalatory strategies:

1. Oppose all proposals and pressures of Iran.

- להתנגד לכל הצעה או לחץ מאיראן.

2. Demonstrate strength in order to intimidate Iran.

להפגין כוח על מנת להרתיע את איראן.

3. Spread negative information about Iran.

להפיץ מידע שלילי אודות איראן.

4. Humiliate and disregard Iran.

להשפיל ולזלזל באיראן.

5. Act as if Israel will never give in, in order to discourage Iran.

לפעול כאילו ישראל לעולם לא תיכנע , כדי להרפות את ידי איראן.

***Glorification***

On the next page, we are interested in how you view the role of Israel in the world.

**בעמוד הבא, אנו מעוניינים להבין כיצד את רואה את תפקידה של ישראל בעולם**

1. All Israelis should respect the customs, the institutions, and the leaders of Israel.

**כל הישראלים צריכים לכבד את המנהגים, המוסדות והמנהיגים הישראלים.**

2. Relative to other nations, we are a very moral nation.

**יחסית למדינות אחרות, ישראל היא מדינה מאוד מוסרית.**

3. Other nations can learn a lot from us.

**מדינות אחרות יכולות ללמוד דברים רבים מאתנו.**

***Demographic Information***

Almost done!

Please answer the following questions about yourself. This information will only be used for statistical purposes.

Remember, all your responses are confidential.

תכף מסיימים !

השאלות הבאות הינן שאלות הנוגעות לנתונים אודותיך .

שימוש במידע זה הינו למטרות סטטיסטיות בלבד.

זכור, כי כל תשובותיך במחקר הינן חסויות.

***Age:***

מהו גילך?____

***Gender***

מהו מגדרך?

Man =   גבר

Woman =  אישה

***Education***

What is the highest level of education you have completed?

מהי ההשכלה הגבוהה ביותר שרכשת ?

יסודית Elementary School

תיכונית high school

על תיכונית	Higher education

תואר ראשון bachelor's degree
תואר שני master’s degree

דוקטורט Doctoral degree

***Political Orientation***

**Please answer the following questions about your political orientation by moving the slider below.**

In general, I am...

  באופן כללי אני....

 ימני -   right wing

שמלאני -   left wing
